# Smith County Medical Society

**Published:** 1891-12

**Authors:** 


### Society Notes.

Smith County Medical Society, sixth regular bi-monthly meeting, held in Tyler, December 8. Annual election of officers. The officers chosen for the ensuing year are—President Dr. A. P. Baldwin; Vice-President, Dr. A. L. Montgomery; Secretary, Dr. Walter Scott; Treasurer, Dr. Z. F. Lillard. No other business was transacted—the papers prepared for this meeting will be presented at the next regular session, February 9, 1892, (second Tuesday.) The retiring officers are—President, Dr. E. R. W. McCreary, of Hopewell, and Dr. J. Z. Ferrell, Tyler, Secretary. To the latter gentleman the JOURNAL is indebted for the above report.

The Smith County Society is a progressive, hardworking and earnest set of physicians, who appreciate the necessity of study, and of frequent consultations, such as are afforded by these meetings, and the advantages arising therefrom. They invite all their progressive brethren of the county and vicinity to meet with them and share the benefit of their meetings.

The Ladies' Home Journal.—As many doctor's wives read DANIEL'S TEXAS MEDICAL JOURNAL (as we have very often been assured), we call their attention to a publication which we are sure will interest them more than our dry discussions of medical topics—the *Ladies' Home Journal.* A sample copy strayed into our office, and our daughters were so charmed with it that at their solicitation we arranged to have it sent regularly for their benefit. It *is just splendid.* It is published in Philadelphia, by the Curtis Publishing Company, and is *only one dollar a year.*

At the head of the editorial department are many of the most distinguished women of America, and many celebrated men—all well known writers. The number and variety of interesting subjects it contains is remarkable, and the *Journal* is handsomely illustrated;—the Christmas number especially, being a *gem.* Under a beautiful new cover, the December number is a model. Dickens' favorite daughter, Mamie, makes her debut as a writer in this number. Amelia E. Barr tells about when she was a girl, Mrs. Harrison, the clever author of the "Auglomaniacs," describes social life in New York—(and who more competent to do it?) Rebecca Cameron describes Christmas on an old plantation —while from across the ocean come Christmas greetings to American women from Aelina Patti, Edna Syall, Sady McKinzie, and other distinguished *literati*, and hosts of American authors of note contribute to make the *Ladies' Home Journal* a *none such.* Send for sample and mention this article.

